# Thoracoabdominal Aortic Replacement Together with Curative Oncological Surgery in Retroperitoneal and Spinal Tumours

**DOI:** 10.3390/curroncol30030195

**Published:** 2023-02-21

**Authors:** Brigitta M. Lutz, Klaus-Dieter Schaser, Jurgen Weitz, Johanna Kirchberg, Hagen Fritzsche, Alexander C. Disch, Albert Busch, Steffen Wolk, Christian Reeps

**Affiliations:** 1 Department of Visceral, Thoracic and Vascular Surgery, University Hospital Carl Gustav Carus, Technische Universitaet Dresden, Fetscherstr. 74, 01307 Dresden, Germany; 2 University Center for Orthopedics, Trauma and Plastic Surgery, University Hospital Carl Gustav Carus, Technische Universitaet Dresden, 01307 Dresden, Germany; 3 National Center for Tumor Diseases (NCT/UCC), Faculty of Medicine and University Hospital Carl Gustav Carus, Technische Universitaet Dresden, German Cancer Research Center (DKFZ), Helmholtz-Zentrum Dresden-Rossendorf (HZDR), 01037 Dresden, Germany

**Keywords:** oncovascular surgery, thoracoabdominal aortic replacement, retroperitoneal neoplasms, spinal neoplasms, surgical oncology, individualised treatment, multidisciplinary surgical team

## Abstract

Malignancies with an extended encasement or infiltration of the aorta were previously considered inoperable. This series demonstrates replacement and subsequent resection of the thoracoabdominal aorta and its large branches as an adjunct to curative radical retroperitoneal and spinal tumor resection. Five consecutive patients were enrolled between 2016 and 2020, suffering from cancer of unknown primary, pleomorphic carcinoma, chordoma, rhabdoid sarcoma, and endometrial cancer metastasis. Wide surgical resection was the only curative option for these patients. For vascular replacement, extracorporeal membrane oxygenation (ECMO) was used as a partial left-heart bypass. The early technical success rate was 100% for vascular procedures and all patients underwent complete radical tumour resection with negative margins. All patients required surgical revision (liquor leak, *n* = 2; hematoma, *n* = 3; bypass revision, *n* = 1; bleeding, *n* = 1; biliary leak, *n* = 1). During follow-up (average 47 months, range 22–70) primary patency rates of aortic reconstructions and arterial bypasses were 100%; no patient suffered from recurrent malignant disease. Thoracoabdominal aortic replacement with rerouting of visceral and renal vessels is feasible in oncologic patients. In highly selected young patients, major vascular surgery can push the limits of oncologic surgery further, allowing a curative approach even in extensive retroperitoneal and spinal malignancies.

## 1. Introduction

Due to the late onset of symptoms, retroperitoneal and primary spinal bone tumours are usually advanced at the time of diagnosis and their majority is malignant [1]. In the past, excessive involvement of vascular structures defined the limits of curative surgical resection in malignancies [2]. Especially when reaching the maximum cumulative radiation dose or with inadequate response to chemo- or radiotherapy, surgical irresectability would inevitably result in a palliative situation, even in solitary lesions. Therefore, young and fit patients may be offered an individualised curative approach for tumour entities with excellent oncological results after surgical resection, such as retroperitoneal sarcoma. Reaching good local control by uncompromising wide resections with negative surgical margins is the only real curative option for sarcoma [3]. More aggressive approaches seem to offer lower local recurrence rates and longer survival [4]. Another example is chordoma, a locally aggressive malignant bone tumor, not responsive to chemotherapy and is usually discovered in a late state [5]. Survival strongly depends on local tumor control [6].

Consequently, advanced oncological resection concepts also include vascular resection and replacement of encased or infiltrated peripheral [7] or abdominal arteries [8,9] up to the infrarenal aorta [10,11]. However, the involvement of the aortic thoracoabdominal transition zone, including the visceral and renal arteries, limits radical oncological resections. Replacement of the thoracoabdominal aorta and its branches is already a burdensome surgical procedure for patients. Nevertheless, analogous to open thoracoabdominal aneurysm repair, with the support of extracorporeal membrane oxygenation (ECMO) perfusion, a replacement can be performed in young patients with acceptably low morbidity and mortality [12,13,14] and should also be offered to oncological patients. Therefore, as an interdisciplinary approach, we indicated and performed aortic replacement and debranching of the visceral and renal arteries to enable wide R0 resection (free histologic margin) in solitary malignant disease of the retroperitoneum or spine. Excessive involvement of the aorta and its large branches could thus be treated with curative intention in highly selected cases. This article presents the largest published series of such extensive surgical procedures. The indications, technical feasibility, complications, and results are summarised.

## 2. Materials and Methods

### 2.1. Data Acquisition

The medical records of all patients were reviewed retrospectively, and follow-up data, including computer tomography (CT) and magnet resonance imaging (MRI), were collected and inspected. Only descriptive analyses were used due to the small number of patients. The study was approved by the local ethics committee of Technische Universitaet Dresden, Germany (file reference: BO-EK-225042021).

### 2.2. Patients

**Figure 1:**
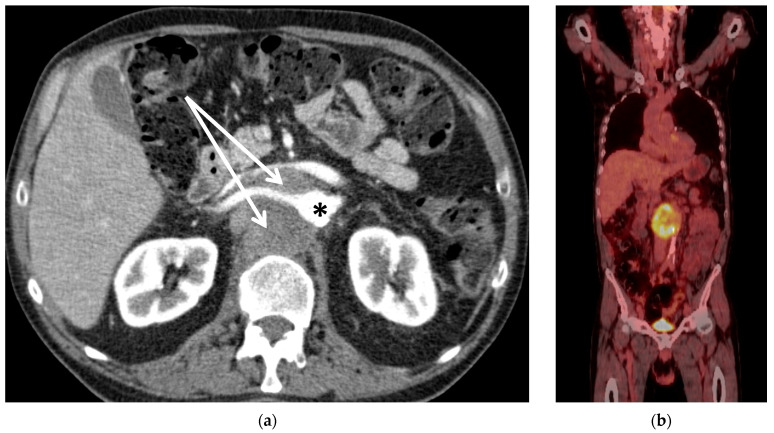
(**a**) CT scan of Patient #1 with retroperitoneal metastasis of unknown primary (arrows) encasing the aorta (*) and right renal artery. The left renal vein is compressed by the tumour mass. (**b**) Corresponding PET-CT scan with FDG-enhancement (yellow) at the site of the tumour and with the absence of further metastasis.

Consecutive patients with the need for wide resection for solitary malignant lesions either encasing or infiltrating the thoracoabdominal aorta were included. For curative surgical treatment, these patients required aortic replacement including visceral and renal branches. Tumour staging was performed by systematic staging with MRI, CT, and fluorodeoxyglucose positron emission tomography (FDG-PET) in all patients. The absence of distant metastasis was confirmed in all patients, using these imaging modalities. The tumour mass of patient #1 can be seen in Figure 1a on the CT scan, and the FDG-PET (Figure 1b) shows that it is a solitary lesion.

CT- or sonography-guided biopsy with histologic workup has been discussed at a multidisciplinary tumour conference. All potential neo-/adjuvant therapy strategies were evaluated and critically discussed, considering the underlying biological behaviour of the malignant tumour, especially responsiveness to radio-, chemo-, or immunotherapy. A curative surgical approach was recommended by the local multidisciplinary tumour board. As an individualised therapy approach, it was seen as the only option for local control with potential long-term survival and for control of immediately threatening tumour-growth-induced complications. Sole palliative care was dismissed due to the young age and good medical condition of all patients with ASA (American Society of Anaesthesiologists physical status) score of I or II and ECOG (Eastern Cooperative Oncology Group) score between 0 and 1 (Table 1). In addition, due to intolerable pain or compression of neighbouring structures, surgery was at least indicated for symptom relief in all cases. Prior to surgery, all patients received detailed interdisciplinary information about the extensive surgical intervention and potential functional limitations, morbidity, and mortality, as well as alternative non-surgical—but less promising—treatment options. Exclusion criteria were the lack of physical fitness, especially with regard to multiple anaesthesias, and the extent of organ sacrifice such as one-sided kidney resection. Further contraindications were multiple metastases or multiple primary lesions.

### 2.3. Standardised Surgical Approach

To reduce surgical trauma and re-establish hemodynamic and clotting homeostasis as well as for recovery of the patient, we used a staged approach and divided major surgical procedures into several sequential steps. Vascular rerouting as a prerequisite for wide retroperitoneal or spinal en bloc resection was always performed first. A standardised approach with a reproducible sequence was used, beginning with the preparation of the left inguinal vessels as access for partial left heart bypass using a peripheral veno-arterial ECMO setup. This was followed by either a transperitoneal or retroperitoneal approach with abdominal or retroperitoneal exploration and definition of oncological resection lines. Subsequently, the splitting of the aortic hiatus exposed the thoracic aorta above the coeliac trunk. Tumour-free zones for visceral and renal artery anastomoses and aortic bifurcation were exposed without opening the tumour compartment. Systemic heparin was administered until reaching an activated-clotting-time of 200 s or more. Retrograde ECMO perfusion was then started for abdominal and retroperitoneal organ and lower limb perfusion in terms of a partial left heart bypass as described by Palombo et al. [12]. The aorta, including the visceral artery segment, was bypassed with a 20 mm tube graft from 8 to 10 cm above the coeliac trunk (end-to-end anastomosis) to the right common iliac artery (end-to-side anastomosis). Visceral and renal bypasses were inserted end-to-side into the main body and end-to-end into the target vessels.

**Figure 2:**
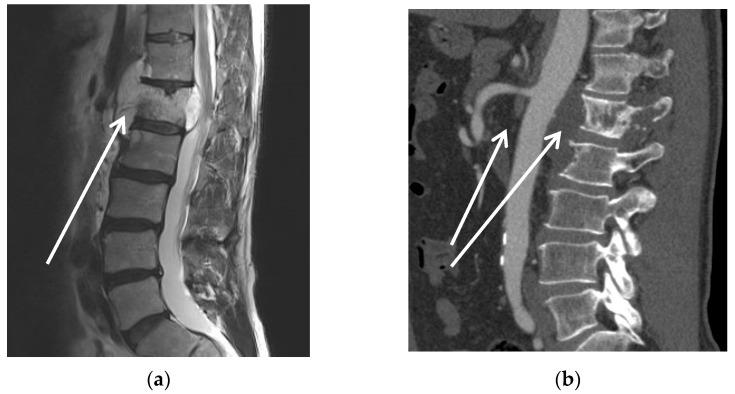

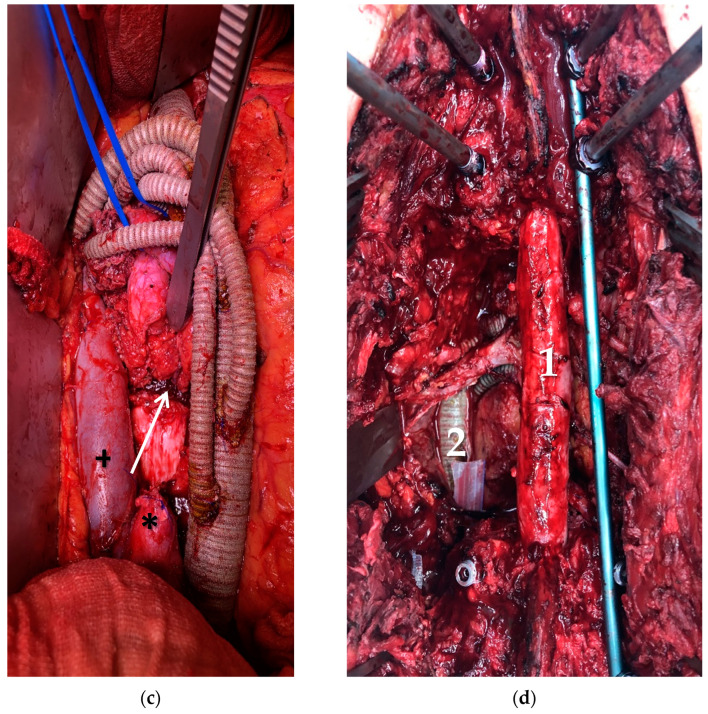
Patient #3; preoperative MRI (**a**) and CT scan (**b**) with arrows indicating tumour of the first lumbar vertebra with encasement of the aorta; (**c**) aorto-iliac bypass with attached bypasses to visceral and renal arteries; vessel loop marks left renal vein, *: aortic stump; +: inferior vena cava; arrow: resected vertebral disc below L2; (**d**) situs after tumour resection from posterior view with partial screw-rod stabilization, completely liberated dural sac (1) after 3-level spondylectomy of vertebrae T12-L2 and aortic prosthesis viewed from the back (2).

Figure 2 shows case #3 with encasement of the aorta, superior mesenteric artery (SMA), and neighbouring structures by chordoma of the first lumbar vertebra. Aortic rerouting can be seen in Figure 2c including the liberation of the anterior aspect of the spine with dissection of the paraspinal muscle insertions and excision of the intervertebral discs (T11/12 and L2/3) as sufficient lateral, cranial, and caudal resection planes.

To reduce the risk of graft infection silver coated polyester prostheses (B. Braun Melsungen AG, Melsungen, Germany) were used for arterial bypasses. The inferior vena cava was replaced with ring-enhanced 20 mm PTFE grafts (W. L. Gore & Associates, Inc., Newark, DE, USA). Non-accessible vessels (inferior vena cava or renal vessels) were addressed in the second step after definitive resection.

**Figure 3:**
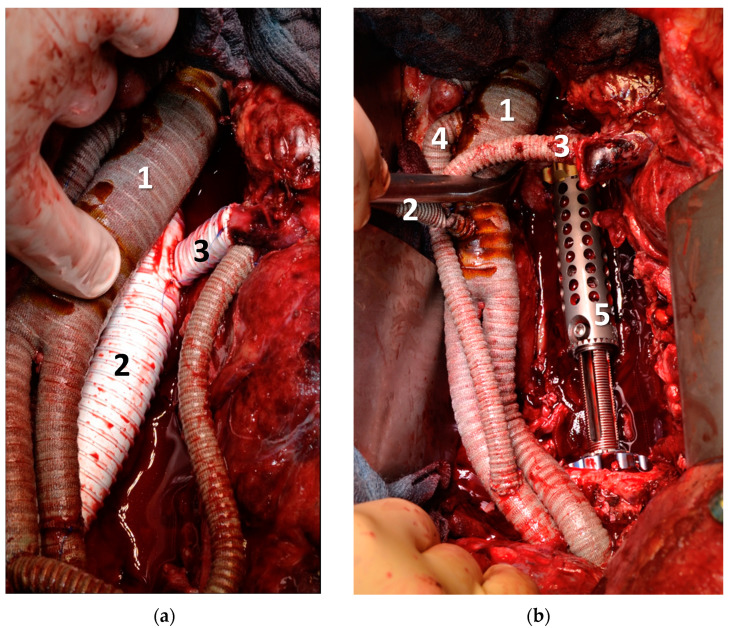
Situs of Patient #1 after resection of retroperitoneal metastasis of unknown primary including the aorta, inferior vena cava, and lumbar vertebrae 1–3; (**a**) aortoiliac bypass (1), and venous ringed bypass (2) with sidebranch to left kidney vein (3), (**b**) aortoiliac bypass (1) with bypass to coeliac trunk (2), superior mesenteric artery (3) and right renal artery (4) and expandable vertebral cage (5).

After the rerouting procedure, all patients were monitored and stabilised in the intensive care unit (ICU). The patients received the thromboprophylaxis dose of unfractionated heparin or low molecular weight heparin starting on day 1 after surgery. In cases with spinal tumour involvement, posterior liberation of the affected vertebrae and internal fixation were performed as the second step. Posterior instrumentation, as depicted in Figure 2d was performed using screw-rod stabilization (DePuy Spine, Raynham, MA, USA). The vertebral column was then replaced with a carbon-composite cage (ostaPek^®^ vertebral body replacement, Coligne AG, Zurich, Switzerland or ObeliscProTM, Ulrich GmbH & Co. KG, Ulm, Germany). Figure 3 shows the arterial and venous rerouting of patient #1 and the 3-level vertebral body replacement with a titanium-expandable cage system.

## 3. Results

Between 2016 and 2020, five consecutive tumour patients (four males, mean age 44 (range 28–56) years) with encasement of the thoracoabdominal aorta including its large branches were treated in our institution. The treated tumour entities were cancer of unknown primary, pleomorphic carcinoma, chordoma, rhabdoid sarcoma, and metastasis of endometrial cancer with no, insufficient, or unsuccessful conservative treatment options. Patient characteristics can be seen in Table 1.

### 3.1. Vascular Procedures

All five patients were treated with the originally intended vascular replacement. The mean operation time was 606 min (range 491–718) for rerouting. Vascular rerouting included replacement of the distal thoracic, complete abdominal aorta in all five patients and further bypasses to the coeliac trunk, superior mesenteric artery, and kidney arteries as can be seen in Table 2. The use of arterio-venous ECMO in all cases reduced the time of organ ischemia to an average of 10 min, the time for the vascular anastomosis between bypass and target vessel. The average total ECMO operating time was 118 min (range 87–144).

### 3.2. Tumour Resection and Adjunctive Procedures

Definitive tumour resection was scheduled for 5.4 days (range 4–8) after the vascular rerouting with a duration of 626 min (range 407–969) for wide tumour resection. In three patients (# 2, 3, and 5), a two-stage approach was used, and in two patients (# 1 and 4), a three-stage approach was used. The entire tumour mass could be resected en bloc in all patients with microscopic negative margins in histopathological workup (*n* = 5). The resected organs can be seen in Table 2. In one patient (#1), resection, and later in situ auto-transplantation of the right kidney were necessary to enable wide resection. In another patient (#4) with complete pancreas resection, portal islet cell transplantation was used to preserve metabolic function as described elsewhere [15].

### 3.3. Outcomes

The mean ICU stay was 44 days (range 20–112). No patient experienced neurologic complications in the sense of spinal ischemia after resection of the thoracoabdominal aorta. Mild paraesthesia of the leg was witnessed in one patient who underwent partial resection of the femoral nerve. All patients showed a transient reduction in their kidney function (glomerular filtration rate) on average 24% (0–57%) after the rerouting and 54% (0–87%) in the first three days after the tumour resection. Three patients (#1, 3, and 5) recovered well after fluid therapy and returned to preoperative kidney function. However, two patients (#2 and 5) developed acute kidney failure requiring haemodialysis. The same two patients had respiratory failure with the need for long-term ventilation, while the other three patients were extubated on the day of the operation or the following day. All patients needed additional surgical procedures due to complications which can be seen in Table 3. The liquor leak was addressed with a dura patch and the cage was realigned under fluoroscopic control. Fluid collection and hematoma were evacuated surgically and the biliary leak was treated by sutures of the common bile duct and percutaneous transhepatic cholangiography with external drainage. The vessel kinking occurred after tumour resection because the graft appeared too long after the tumour was removed and could be corrected with the shortening of the graft. Organ swelling made primary closure of the abdomen unfeasible so patient #5 needed temporary vacuum dressing and later midline closure.

One hepatic bypass was temporarily occluded resulting in a primary patency of 96% and primary-assisted bypass patency of 100% (Table 4). Septic arrosion was observed in one vascular anastomosis at a hepatic artery (#2) and could be controlled with a short graft interposition. In two patients (#2 and 4) with extensive bowel resection, swelling of the intestines led to postponed reconstruction. These two patients died in the hospital due to septic complications, resulting in a 30-day mortality of one and in-hospital mortality of two.

### 3.4. Follow-Up

All three discharged patients survived without recurring tumour disease (mean follow-up: 47 months, range 22–70) and patent bypasses (Table 4). Two patients could walk without any constraints, and one patient needed walking aids for longer distances. One patient (#1) required revision of the posterior instrumentation of the spine after two and three years due to implant failure and one patient (#3) required talc pleurodesis three months after resection due to chylothorax.

## 4. Discussion

This study demonstrates the surgical feasibility, excellent technical success rate, and oncological-prognostic usefulness of wide resection of retroperitoneal or spinal tumours with thoracoabdominal aortic replacement in a heterogeneous series of five oncological patients. Even as independent procedures, open thoracoabdominal aortic repair, and multivisceral wide resection are extensive and technically demanding surgical interventions. The combination of both is even more complex and challenging and is thus prone to complications for the patient. Therefore, patients are most often either rejected and considered inoperable, or treated by intralesional procedures with both options leaving them with palliative treatment and no chance of long-term survival. However, cancer patients with extensive but solitary tumour lesions are candidates for such combined interventions as the only curative option. With optimal local and systemic tumour control by complete resection of the malignant retroperitoneal tumour, these patients have a favourable survival prognosis, because local control is the key to curing primary retroperitoneal and spinal malignancies such as sarcoma [16] and chordoma [6]. The completeness of the resection determines progression-free and overall survival in sarcoma [3,4], isolated colorectal metastasis [17,18], and retroperitoneal recurrences of gynaecologic tumours [19]. Consequently, for patients in good general condition together with clinically relevant tumour symptoms (Table 1), this combination therapy should be considered in specialised centres. Availability of shared experience among orthopaedic, vascular, and visceral surgeons and proceeding as one multidisciplinary team holds the key to technical success and is the mandatory prerequisite to offering a curative surgical approach to such selected patients. This study cohort included individuals with extensive but solitary malignancies in the retroperitoneum, i.e., absence of distant metastasis, as assessed by whole-body PET-CT scan. Biopsy with a histologic classification of the tumour was a prerequisite to distinguish tumour entities with a favourable chance for cure by resection. All cases were discussed by the multidisciplinary tumour board and non-surgical or radio-oncological treatment options alone did not offer curative approaches in any of the included patients. Resection was considered the only potentially curative option in an individualised setting.

In recent decades, the sole presence of vascular involvement was no longer seen as a contraindication for wide resection [7,9,11]. Oncological outcomes after resection of retroperitoneal malignancies were comparable to cases without vessel involvement [20]. Moreover, with the assistance of a partial left heart bypass provided by extracorporeal membrane oxygenation (ECMO), the adverse effects of long organ ischemia during thoracoabdominal rerouting and arterial debranching can be eliminated with limited functional constraints for the patient. Therefore, aortic resection and replacement can be considered even in patients with an extensive disease requiring complex vascular rerouting and revascularization. In our institution, the use of ECMO is now regularly used when thoraco-abdominal aortic replacement is performed. We used ECMO as a partial left-heart bypass instead of a cardiopulmonary bypass (CPB) conventionally used in cardiac surgery because no circulatory arrest was needed and aortic cross-clamping was located below the supraaortal vessels. The ECMO does not have a reservoir, hence no blood stasis is present and less anticoagulation with heparin is needed. Usually, CPB needs an activated clotting time (ACT) of around 480 s. whereas for ECMO use, only mild heparinization is needed. We used an ACT of around 200 s. while the ECMO was running and vessels were clamped or blocked. With this moderate use of heparin, we expected a good balance between anticoagulation during vessel replacement and haemostasis so that the subsequent tumor resection did not cause too much bleeding.

Wide resections for retroperitoneal tumours involving both vascular and spinal structures, as performed by vast aortic resection and multilevel en bloc spondylectomy followed by vertebral body replacement, is remarkable. Compared with the present literature, the extent of vascular rerouting, including the thoracoabdominal aorta and visceral- and renal arteries, and the use of temporary ECMO in this series is unique (Table 5).

To date, only four series of various vascular replacements mention the replacement of the thoracoabdominal section of the aorta (Poultsides et al. [20] one thoracoabdominal replacement including two visceral bypasses, Schwarzbach et al. [11] replacement of thoracoabdominal section twice but without visceral and renal branches, Song et al. [21] and Homsy et al. [24] each reporting one thoracoabdominal replacement with bypasses to hepatic artery, SMA and left renal artery), and one case report (Gösling et al. [22] and Graulich et al. [23]; same case with a thoracoabdominal aortic replacement but without visceral or renal rerouting). To our knowledge, this is the largest published series of combined thoracoabdominal aortic and visceral resection and reconstruction in patients with vascular and spinal involvement due to retroperitoneal or spinal tumour growth (Table 5). In particular, the extent of aortic resection and the sum of renal and visceral artery reconstructions have not yet been reported. In comparison to the remaining literature, with primary patency of 58–89% [11,20,21,22,23], this series with primary patency of 96% and primary-assisted patency of 100% of all vascular reconstructions had excellent results (Table 5).

Owing to the extent of the combined surgery, all patients required surgical revision of any kind, mostly linked to radical tumour resection. Only one patient had vascular complications with occlusion of a visceral bypass and later bleeding due to septic arrosion. None of the patients had paraplegia, even though large parts of the aorta including essential thoracic and lumbar segmental arteries or multiple spinal levels with epidural tumour compression were resected. Resection of these vital segmental arteries has already been proven to be safe in orthopaedic resections [25].

The prior use of chemo- or radiotherapy might have had both benefits and disadvantages. As a benefit of neo-adjuvant treatment, resection margins were probably thicker or would involve more different tissues. The presumably more radical resections might support the goal of cure due to uncompromising local control. At the same time, neo-adjuvant treatment with consecutive scaring might lead to injury of essential structures during preparation. Especially if scar tissue makes it difficult to optically discriminate neighbouring structures or if it is technically demanding to dissect adhering structures, intestinal or nerve damage is possible. Further, prior therapy might lead to prolonged healing time, leading again to further issues such as leaking intestinal anastomosis or open wound healing with possible bacterial contamination of bypass or internal fixation material. The relevant hospital mortality of two out of five in this study needs to be discussed. Certainly, it is obvious that the combination of two maximum surgical interventions within a short time interval is risky. In particular, the combination of visceral organ resection with prosthetic vascular reconstruction, as well as vertebrectomy and cage reconstruction bears a high risk of septic and bleeding complications. Especially, postponed gastro-intestinal reconstructions because of intestine swelling might further exacerbate septic complications in the sequel. Mortality cannot be compared to the published series mentioned above because of the heterogeneous locations and smaller extent of replaced vessels in the available literature. In series with the replacement of just the infrarenal aorta due to malignant processes, mortality was lower and ranged between 0–17% [26,27]. If related to the mortality of thoracoabdominal replacement for aortic aneurysms of 8–15% [13,28,29], the 30-day mortality of one and in-hospital mortality of two out of five patients seems high. However, the patients in this series bear the inherent burden of additional major tumour operations with relevant intraoperative and postoperative morbidity and mortality [30,31]. Both deceased patients requested surgical therapy, with patient # 2 suffering from constant pain and bowel obstruction due to the large tumour mass. Patient # 4, a medical expert himself, requested surgical therapy with a curative intention for favourable outcomes in sarcoma compared to palliation.

In all patients who survived the intermediate postoperative phase, tumour-free survival was given until the end of follow-up; matching, or even exceeding the clinical results published by other groups (Gösling et al. [22] 100%, Schwarzbach et al. [11] 90% after two years, 67% after five years). Good long-term outcomes allowed patients to return to their daily routines with good mobility and normal bowel and bladder functions. The freedom of recurrent disease of all surviving patients seems promising but needs to be re-evaluated in a longer follow-up period with a larger number of patients.

## 5. Conclusions

Incomplete resection has long been seen as a predictor for poor outcomes in the surgical treatment of retroperitoneal sarcoma [32]. As a consequence radical surgery is indicated even if it takes vascular replacement. Extensive combined major oncovascular surgery must be well prepared with optimal imaging such as CT, MRI, and PET-CT. Biopsy and histopathological examination are obligatory to further clarify the type of malignancy. Distant metastases need to be excluded preoperatively by all means in order to justify the complexity and complication profile of the extensive surgical procedure. A closely cooperating and well-experienced multidisciplinary team should then advise an individualised treatment plan including the indication for surgery. A multimodal approach with neoadjuvant radiotherapy and or chemotherapy should be considered. Both morbidity and mortality need to be discussed in detail with the patient, considering that surgery is the only curative treatment option in highly selected cases. Using a curative approach, reconstruction and resection of the large vessels, especially in the thoracoabdominal segment, has proven feasible and shows good patency. This further expands the limits of surgical resectability in oncological patients towards curative treatment concepts.

### Limitations

This single-centre study consists of a small number of patients as a result of individual treatment recommendations for every case. Thus, this study is descriptive only. It is a retrospective analysis of the patient records. Hence, no preregistration exists for the study reported in this article.

## Figures and Tables

**Table 1:** Patient characteristics; ECOG: Eastern Cooperative Oncology Group score, ASA: American Society of Anesthesiologists physical status classification system, CUP: cancer of unknown primary, L: lumbar vertebra, Gy: Gray.

Pat. No.	Sex	Age	Diagnosis	Presentation	Neo-Adjuvant Radiotherapy	Neo-Adj. Chemo-Therapy	ECOG	ASA
1	m	56	CUP with vertebral destruction L1-3	Intolerable pain in the spine	Yes (50 Gy)	Yes	1	II
2	m	28	Retro-peritoneal pleomorphic carcinoma	Compression of abdominal organs; bowel obstruction and hydronephrosis	Yes (50 Gy)	Yes	0	II
3	m	56	Chordoma L1	Spinal compression with impending paraplegia	Yes, proton therapy (50 Gy)	No	1	I
4	m	41	Retro-peritoneal rhabdoid sarcoma	hydronephrosis; patient’s wish for curative resection	Yes, proton therapy (50 Gy)	Yes	0	II
5	f	40	Retro-peritoneal metastasis of endometrial carcinoma	Incipient nerve infiltration at the left femoral nerve	No further radiotherapy possible	No	1	II

**Table 2:** Vessel replacement and organ resection.

	Patient #					
	1	2	3	4	5	Total
**Vessels replaced:**						
thoraco-abdominal aorta	1	1	1	1	1	**5**
coeliac trunk		1	1	1	1	**4**
common hepatic artery	1					**1**
superior mesenteric artery	1	1	1	1	1	**5**
renal artery	2	1	2	1	1	**7**
inferior vena cava	1	1		1	1	**4**
**Resection:**						
kidney (unilateral)		1		1	1	**3**
3-level spondyl-ectomy	1		1			**2**
ventral parts of the vertebral body		1		1		**2**
inferior vena cava	1	1		1	1	**4**
pancreas		1		1		**2**
stomach		1		1		**2**
left hemicolon		1		1		**2**
diaphragm (partial)		1		1	1	**3**
femoral nerve (partial)					1	**1**
aorta	1	1	1	1	1	**5**

Bold numbers are the sum of all patients.

**Table 3:** Postoperative course with the need for revision surgery.

Postoperative Course:	Patient #					
	1	2	3	4	5	Total
liquor leak	1		1			**2**
cage dislocation	1					**1**
fluid collection	1					**1**
biliary leak		1				**1**
hematoma		1		1	1	**3**
bypass occlusion		1				**1**
anastomotic bleeding		1				**1**
vessel kinking				1		**1**
open abdomen					1	**1**

Bold numbers show the total amount in the cohort.

**Table 4:** Bypass patency and follow-up (n/a: not applicable).

#	Bypass Patency	Death (Reason)	Follow Up	Recurrent Disease
			(in Months)	
1	100% primary	No	65	no
2	occlusion of hepatic bypass, secondary patency 100%	Yes (septicemia)	0	n/a
3	100% primary	No	44	no
4	100% primary	Yes (septicemia)	0	n/a
5	100% primary	No	21	yes

**Table 5:** Literature review of thoracoabdominal aortic replacement in oncological patients (TRC: coeliac trunk, LRA: left renal artery, RRA: right renal artery, SMA: superior mesenteric artery).

Author	Year	Treated Malignancies	Number of All Patients	Number of Vessel Replacements:				In Hospital Mortality	Reported Patency	Resection with Negative Margin	Survival	Freedom of Recurrent Disease
				Thoracoabdominal	Renal Artery	Visceral Artery	Reimplantation					
Schwarz- bach et al. [11]	2006	retroperitoneal soft-tissue sarcoma	25	2	0	0	1 (TRC)	4% of the whole series	primary: 88.9% of the whole series	40% in the whole series	2 years: 50%, 5 years: 38%	not specified
Song et al. [21]	2009	retroperitoneal or extremity sarcoma	14	1	0	0	3 (LRA, SMA, hepatic artery)	0	primary 58% and assisted 83% of the whole series	43% of Retroperitoneal Sarcomas	5-year (overall): 68%, 5-year (disease-free): 52%	79%
Goesling et al. [22] and Graulich 2019 et al. [23] (same case)	2013/2019	chondrosarcoma	1	1	0	0	0	0	100%	100%	100% in 12 years	100%
Poultsides et al. [20]	2015	Sarcoma, not further specified	50	1	1	2 (SMA and hepatic artery)	0	2% of the whole series	primary 86% and assisted 92% of the whole series	76%	5 year (overall): 59%	49%
Homsy et. al. [24]	2021	miscellaneous, different locations	17	1	1	2	0	0	patency was not reported for all patients	56%	61% in 3 years	46% in 3 years
current series, Lutz et al.	2023	miscellaneous, all located in retroperitoneal space	5	5	7 (2 LRA, 5 RRA)	9 (3 TRC, 5 SMA, 1 hepatic artery)	1 (TRC)	2 out of 5	primary 96%, assisted 100%	100%	60% overall and disease-free, (mean follow-up 47 months)	100%

## Data Availability

The data presented in this study are available in the article.
